# “Low-risk groups” deserve more attention than “high-risk groups” in imported COVID-19 cases

**DOI:** 10.3389/fmed.2023.1293747

**Published:** 2023-11-30

**Authors:** Wanshan Zheng, Ying Tan, Zedi Zhao, Jin Chen, Xiaomei Dong, Xiongfei Chen

**Affiliations:** ^1^Department of Public Health and Preventive Medicine, School of Medicine, Jinan University, Guangzhou, Guangdong, China; ^2^Guangzhou Center for Disease Control and Prevention, Guangzhou, Guangdong, China

**Keywords:** COVID-19, imported case, quarantine period, time interval, risk factor

## Abstract

**Objective:**

To estimate the optimal quarantine period for inbound travelers and identify key risk factors to provide scientific reference for emerging infectious diseases.

**Methods:**

A parametric survival analysis model was used to calculate the time interval between entry and first positive nucleic acid test of imported cases in Guangzhou, to identify the influencing factors. And the COVID-19 epidemic risk prediction model based on multiple risk factors among inbound travelers was constructed.

**Results:**

The approximate 95th percentile of the time interval was 14 days. Multivariate analysis found that the mean time interval for inbound travelers in entry/exit high-risk occupations was 29% shorter (OR 0.29, 95% CI 0.18–0.46, *p* < 0.0001) than that of low-risk occupations, those from Africa were 37% shorter (OR 0.37, 95% CI 0.17–0.78, *p* = 0.01) than those from Asia, those who were fully vaccinated were 1.88 times higher (OR 1.88, 95% CI 1.13–3.12, *p* = 0.01) than that of those who were unvaccinated, and those in other VOC periods were lower than in the Delta period. Decision tree analysis showed that a combined entry/exit low-risk occupation group with Delta period could create a high indigenous epidemic risk by 0.24.

**Conclusion:**

Different strata of imported cases can result in varying degrees of risk of indigenous outbreaks. “low-risk groups” with entry/exit low-risk occupations, fully vaccinated, or from Asia deserve more attention than “high-risk groups.”

## Introduction

On March 11, 2020, the World Health Organization (WHO) declared that the coronavirus disease 2019 (COVID-19) had developed into a global pandemic (1), and by August 9, 2023, approximately 769 million COVID-19 cases have been reported globally, with a cumulative total of 6.954 million deaths (2), and the majority of countries have reported COVID-19 case-fatality rates ranging from 0.5 to 5.0% (3). During the outbreak, inbound travelers are a high-risk group for potential infection. Therefore, it is crucial to defend externally against importation.

Guangzhou is a pivotal city in epidemic prevention and control of imported cases in China. Investigations showed that approximately 20,000 inbound travelers were under quarantine and observation in Guangzhou each day during the COVID-19 period. Even though China has announced a roll-back of its strict anti-COVID-19 measures at this stage, against the background of a certain percentage of infected inbound travelers, it is still important for inbound travelers to take proper quarantine measures and self-monitoring for a while after entry. However, a study by Bai et al. published in JAMA found that it took 19 days for a close contact to test positive for nucleic acids and may have resulted in transmission including five people (4). Therefore, it is necessary to effectively manage quarantine of close contacts and monitor the risk groups of disease after the quarantine period has ended.

Model parameter estimation based on survival analysis (5) can better estimate the time interval between entry and the first positive nucleic acid test, incubation period (6), or latent period for infectious diseases, and also serve as a valuable validation and supplement to commonly known quarantine periods. This paper estimated the time distribution characteristics of imported cases from entry to the first nucleic acid positivity and identified the influencing factors through survival analysis. Additionally, a decision tree model was used to construct the influencing factors-based outbreak risk prediction model for inbound travelers (7), which can help to predict, evaluate and follow up the risk of an indigenous epidemic caused by inbound key travelers. It could explore effective methods of balancing healthcare costs and socio-economic benefits (8) while controlling the spread of epidemic outbreaks, and it can provide scientific references for the prevention and control of emerging infectious diseases.

## Materials and methods

### Data sources and study population

Data on the imported COVID-19 epidemics in Guangzhou were obtained from the official website of the Guangzhou Health Commission and the notification of imported epidemics published by the Guangzhou Center for Disease Control and Prevention as of twenty-four o’clock on August 31, 2022. The following were excluded from the study: (1) duplicate cases; (2) cases with excessive missing information; and (3) native cases. A total of 1,029 imported COVID-19 cases were collected in this study, and after cases with missing test data from entry to the first nucleic acid positivity were excluded, a total of 743 cases were used to calculate the survival analysis. This study was reviewed by the Institutional Review Board of the Guangzhou Center for Disease Control and Prevention.

### Research method

Retrospective collection of information on reported inbound cases (confirmed cases and asymptomatic patients) in Guangzhou from March 1, 2020, to August 31, 2022, and extraction of demographic characteristics such as age, gender, occupation, and nationality of the patients, as well as information on their entry into China, history of sojourn, history of COVID-19 vaccination, and information on nucleic acid testing.

### Measurements and definition

(1) Quarantine period: The quarantine period refers to the duration of the quarantine. Counting from the last day of contact with the patient, the quarantine period for the contact is established according to the longest incubation period of the disease. During the quarantine period the contact is placed under medical observation, retained for examination or other necessary measures, (2) Confirmed cases (9): patients who met the relevant epidemiologic history, clinical manifestations, and tested positive for COVID-19 nucleic acid by real-time fluorescence RT-PCR, (3) Asymptomatic patients: no clinical symptoms and respiratory specimens with positive COVID-19 pathogenicity test, (4) Imported cases (imported asymptomatic patients): cases with a history of residence in an outbreak country or region within 14 days before the positive nucleic acid test, and exclude infection in China, (5) The time interval between entry and the first positive nucleic acid test: the starting time of observation was the entry time of each study subject, with the first positive nucleic acid test result as the outcome event, and the survival time was defined as the interval from entry to the first positive nucleic acid test. Assuming that E and S are the entry time of a case and the time point at which a case’s nucleic acids can be first detected as positive, respectively, the time interval between entry and the first positive nucleic acid test is T = S−E. When both E and S fall on limited intervals, the observation is called the doubly interval-censored data. Accordingly, when one of E and S is an exact value and the other falls on a limited interval, the observation is called the singly interval-censored data. When both E and S are exact values, the observed data is the fully data (10). The time of entry was determined for all cases in this study, and the time point at which nucleic acids could be first detected as positive fell on a limited interval, so the observations were singly interval-censored data, i.e., T = (S_L_−E, S_R_−E), where the subscripts L and R denote the time of the last negative before a positive nucleic acid result, and the time of the first positive result (i.e., the lower and upper limits), respectively. When the interval between the positive result and the last negative result before the positive result was less than or equal to 1 day (performed with a time distance of 24 h) (11), both were considered to have occurred on the same day, i.e., L = R, then the time interval between entry and the first positive nucleic acid test (T) is fully data. In contrast, data were interval-censored if the time interval between the positive result and the last negative result was greater than 1 day because L did not equal R.

### Statistical analysis

Statistical analysis was performed using R4.0.4 and SAS 9.4 software. Epidemiological characteristics of imported COVID-19 cases were analyzed using descriptive methods, count data were expressed as frequency, constitutive ratio, or proportion (%), and differences between groups were analyzed using the chi-square test or Fisher’s exact probability method. Differences were considered statistically significant at *p* < 0.05.

Distributional and point estimates of the time interval between entry and the first positive nucleic acid test for imported cases were based on a parametric survival analysis model of the failure data (12, 13). The model is $\log\left(T\right)=\mu +\sigma \log\left(T_{0}\right)$ if there is no covariate, or $\log\left(T\right)=x^{,}\beta +\sigma \log\left(T_{0}\right)$ if there is a covariate. Where T represents the failure time, μ represents the location parameter, σ represents the scale parameter, $T_{0}$ represents some sample that is in the baseline distribution, and x represents the value vector of the covariates. The model was fitted using the parametric method; the distributions commonly used for T are the Weibull distribution, the Log-normal distribution, etc.; the maximum likelihood method was used for the fitting, and the log-likelihood function was:


$$\begin{array}{l} L=\sum \log(f\left({\bar{\omega }}_{i}\right)/\sigma +\sum \log\left(S\left({\bar{\omega }}_{i}\right)\right)+\sum \log\left(F\left({\bar{\omega }}_{i}\right)\right)\\ \;+\sum \log\left(F\left({\bar{\omega }}_{i}\right)-F\left(v_{i}\right)\right)\text{.} \end{array}$$

Where the first term of the formula sums over no censored data, the second term sums over right-censored data, the third term sums over left-censored data, and the last term sums over interval-censored data.

The covariate-free model was first used to fit the model using Log-normal, Log-logistic, Weibull, and Gamma distributions, respectively, to estimate the parameters of the time interval between entry and the first positive nucleic acid test and their distributional characteristics for each distribution, and to determine the fitted optimal distribution based on the maximum likelihood method and Bayesian Information Criterion; The distribution was then applied to a univariate covariate model analysis to analyze the individual effects of each factor on the time interval between entry and the first positive nucleic acid test, and those factors with *p* < 0.05 in the univariate analysis were entered into the multivariate analysis. Afterward, the factors that were statistically significant in the multivariate analysis were analyzed in subgroup analysis to estimate their percentile at some critical time of the time interval between entry and the first positive nucleic acid test.

The degree of risk of an indigenous outbreak due to imported cases was defined as follows: the time interval between entry and the first positive nucleic acid test ≤ P95 as low risk; > P95 as high risk. A decision tree model was used to combine statistically significant variables from the univariate analysis with the outbreak data to construct a dataset for the COVID-19 outbreak risk simulation model for Guangzhou City. Utilizing 70% of the data for training purposes and 30% for testing, a decision tree is built using the classification and regression trees (CART) algorithm (14), which can rank the importance of the influencing factors and construct a multi-risk factor-based simulation model of COVID-19 outbreak risk for inbound travelers in Guangzhou City.

## Results

### Baseline information for imported cases

In this study, 520 (50.53%) of the 1,029 imported cases were asymptomatic patients, and 509 (49.47%) were confirmed cases. The majority of imported cases were male (67.54%) and young adults (52.67%), and there were more imported cases from Asia (78.23%) and foreign nationalities (63.75%). Entry/exit high-risk occupational groups refer to those who work in key areas such as border crossings with a high risk of disease transmission, including crew members, seafarers, logistics personnel at airports and customs, and other occupational groups closely related to entry/exit. These groups accounted for 28.09% of the population. Imported cases from different VOC periods were dominated by the Omicron period, which accounted for 61.80% of the total. In addition, there were differences in the composition of asymptomatic patients and confirmed cases in terms of age, import source, nationality, and occupation, with Chinese nationals predominantly confirmed cases and foreigners predominantly asymptomatic patients (Table 1).

**Table 1:** Basic information on imported COVID-19 cases from abroad in Guangzhou.

Variable category	Aggregate *N* = 1,029 *n* (%)	Asymptomatic patients *N* = 520 *n* (%)	Confirmed cases *N* = 509 *n* (%)	*p* values
Sex				
Men	695 (67.54)	360 (51.80)	335 (48.20)	0.27
Women	334 (32.46)	160 (47.90)	174 (52.10)	
Age				
<18	64 (6.22)	34 (53.13)	30 (46.88)	<0.001
18~	542 (52.67)	300 (55.35)	242 (44.65)	
40~	381 (37.03)	176 (46.19)	205 (53.81)	
≥65	42 (4.08)	10 (23.81)	32 (76.19)	
Region				
Asia	805 (78.23)	376 (46.71)	429 (53.29)	<0.001
Africa	91 (8.84)	54 (59.34)	37 (40.66)	
Oceania	57 (5.54)	46 (80.70)	11 (19.30)	
North America	39 (3.79)	20 (51.28)	19 (48.72)	
Europe	34 (3.30)	22 (64.71)	12 (35.29)	
South America	3 (0.29)	2 (66.67)	1 (33.33)	
Nationality				
Chinese	361 (35.08)	92 (25.49)	269 (74.52)	<0.001
Hong Kong, Macau, and Taiwan	12 (1.17)	5 (41.67)	7 (58.33)	
Foreign	656 (63.75)	423 (64.48)	233 (35.52)	
Entry/exit risk occupations				
High	289 (28.09)	120 (41.52)	169 (58.48)	<0.001
Low	740 (71.91)	400 (54.05)	340 (45.95)	
International student				
Yes	85 (8.26)	61 (71.76)	24 (28.24)	<0.001
No	944 (91.74)	459 (48.62)	485 (51.38)	
Background diseases				
Yes	55 (5.34)	19 (34.55)	36 (65.46)	<0.001
No	784 (76.19)	426 (54.34)	358 (45.66)	
Unknown	190 (18.46)	75 (39.47)	115 (60.53)	
Vaccination doses				
Unvaccinated	322 (34.77)	196 (60.87)	126 (39.13)	<0.001
1 dose	64 (6.91)	40 (62.50)	24 (37.50)	
2 doses	256 (27.65)	102 (39.84)	154 (60/16)	
3 or more doses	284 (30.67)	144 (50.70)	140 (49.30)	
VOC periods				
Alpha	188 (18.41)	128 (68.09)	60 (31.91)	<0.001
Beta	34 (3.33)	19 (55.88)	15 (44.12)	
Gamma	32 (3.13)	15 (46.88)	17 (53.13)	
Delta	136 (13.32)	74 (54.41)	62 (45.59)	
Omicron	631 (61.80)	283 (44.85)	348 (55.15)	

### Covariate-free survival analysis results

The Log-normal, Log-logistic, Weibull, and Gamma distributions were employed to approximate the time interval between entry and the first positive nucleic acid test in Guangzhou. The optimal distribution model was determined through the Bayesian information criterion (BIC) or the maximum likelihood method (15). The log-likelihood ratio (LLR) and Bayes calculated from both the Log-normal and Log-logistic distributions were relatively close, and the location parameter estimates were generally similar. However, the Weibull and Gamma distributions estimate model parameters that were not statistically significant. Moreover, given that the Log-normal distribution has a lower likelihood ratio and BIC compared to the Log-logistic distribution, and since it is more commonly used in estimating latency distributions, it is considered to be the optimal choice for analyzing the time interval between the entry and the first positive nucleic acid test for COVID-19. The mean time interval between entry and the first positive nucleic acid test estimated by the Log-normal distribution was 0.4 days with a 95% confidence interval of (0.32, 0.51) days, which means that 50% of the imported cases tested positive within 1 day of entry. In addition, approximate 95% (94.51%) of the imported cases first tested positive within 14 days of entry (Table 2).

**Table 2:** Survival analysis results of four distribution parameter models.

Distribution form	LLR	BIC	Location parameter			The mean time interval between entry and the first positive nucleic acid test		95th percentile (day)	7-day centile	14-day centile
			Estimate	95% CI	*p* values	estimate	95%CI			
Log-normal	−1001.06	2015.33	−0.90	(−1.14, -0.67)	<0.0001	0.40	(0.32,0.51)	15.52	90.11%	94.51%
Log-logistic	−1018.26	2049.74	−0.82	(−1.04, -0.61)	<0.0001	0.44	(0.35,0.55)	20.35	89.36%	93.45%
Weibull	−989.56	1992.33	0.10	(−0.08, 0.29)	0.28	0.51	(0.41,0.63)	11.43	90.84%	96.31%
Gamma	−988.88	1997.59	−0.11	(−0.50, 0.29)	0.59	0.48	(0.34,0.67)	11.89	90.72%	96.01%

### Univariate survival analysis results of influencing factors

Log-normal distribution has been employed in the univariate survival analysis. In total, various aspects such as sex, age, region, nationality, entry/exit risk occupation, international student status, background disease condition, vaccination history, and VOC period were investigated and analyzed. The results indicated that there was a statistically significant difference for the factors of entry/exit high-risk occupations (*p* < 0.0001), Oceania (*p* = 0.02), Africa (*p* < 0.0001), COVID-19 vaccination with 2 doses (*p* < 0.0001), COVID-19 vaccination with 3 or more doses (p < 0.0001), Alpha period (*p* < 0.0001), Beta period (*p* < 0.0001), and Omicron period (*p* = 0.0001) (Table S1).

### Multivariate survival analysis results of influencing factors

Factors with *p* < 0.05 in the univariate survival analysis were calculated in the multivariate analysis, which found that the mean time interval between entry and the first positive nucleic acid test for inbound travelers in entry/exit high-risk occupations was 29% shorter (OR 0.29, 95% CI 0.18–0.46, *p* < 0.0001) than that of low-risk occupations, those from Africa were 37% shorter (OR 0.37, 95% CI 0.17–0.78, *p* = 0.01) than those from Asia, those who received 3 or more doses of the COVID-19 vaccinations were 1.88 times higher (OR 1.88, 95% CI 1.13–3.12, p = 0.01) than that of those who did not, and those in other VOC periods were lower than in the Delta period (Table 3).

**Table 3:** Multivariate survival analysis of factors influencing the time interval between entry and the first positive nucleic acid test of COVID-19.

Variable category		References	B	SE	p values	OR	OR 95%CI
Entry/exit risk occupations	High	low	−1.23	0.23	<0.0001	0.29	(0.18, 0.46)
Region	Oceania	Asia	0.53	0.31	0.09	1.70	(0.93, 3.14)
	Africa		−1.00	0.38	0.01	0.37	(0.17, 0.78)
Vaccination doses	2 or more doses	unvaccinated	0.38	0.24	0.11	1.46	(0.92, 2.36)
	3 or more doses		0.63	0.26	0.01	1.88	(1.13, 3.12)
VOC periods	Alpha	Delta	−3.07	0.35	<0.0001	0.05	(0.02, 0.09)
	Beta		−4.73	1.05	<0.0001	0.01	(0.00, 0.07)
	Omicron		−1.30	0.24	<0.0001	0.27	(0.17, 0.44)

### Subgroup analysis

The study established the time interval between entry and the first positive nucleic acid test as 14 days, which can inform the quarantine period for inbound travelers. Therefore, we focused on the 14-day centile, which was defined as the proportion of imported cases with a positive first nucleic acid test within 14 days of entry. By calculating the 14-day centile for different factors, the proportion of different types of inbound travelers who did not test positive until after the end of the quarantine period can be derived, thus identifying priority risk groups. The factors with statistical significance in the multivariate survival analysis were entered into subgroup analysis, and the 14-day centile of the time interval between entry and the first positive nucleic acid test was calculated. The results found that 6.52% of entry/exit low-risk occupational cases first tested positive 14 days after entry, which was higher than the proportion of high-risk occupational cases; 5.44% of Asian cases tested positive 14 days after entry, whereas basically all African cases could be detected as positive within 14 days of quarantine; and the proportion of cases who had received 3 or more doses of the COVID-19 vaccinations was 8.18%, which was far higher than the proportion of unvaccinated cases. As the Guangzhou COVID-19 Nucleic Acid Detection System was not fully standardized until May 2021, many of the nucleic acid positive detection times for imported cases in Guangzhou prior to May 2021 were missing. Consequently, the mean time interval between entry and the first positive nucleic acid test result in the Alpha and Beta periods was lower than that in the Delta period during multivariate analyses, possibly due to a lack of representative samples. As a result, subgroup analyses were only conducted for the Delta and Omicron periods, which mainly occurred after May 2021. The analysis found that 12.50% of imported cases in the Delta period first tested positive 14 days after entry, while 5.50% of imported cases in the Omicron period were tested 14 days after entry (Table 4).

**Table 4:** Subgroup analysis of the time interval between entry and the first positive nucleic acid test of COVID-19.

Variable category	14-day centile	Percentage of positive detections after 14 days of quarantine
Entry/Exit low-risk occupations	93.48%	6.52%
Entry/Exit high-risk occupations	98.12%	1.88%
Asia	94.56%	5.44%
Africa	99.26%	0.74%
Unvaccinated	97.73%	2.27%
3 or more doses of COVID-19 vaccinations	91.82%	8.18%
Delta	87.5%	12.50%
Omicron	94.5%	5.50%

### Decision tree analysis

Cases detected within the quarantine period have a lower risk of causing an indigenous outbreak so the degree of risk of an indigenous outbreak due to imported cases was defined as follows: the time interval between entry and the first positive nucleic acid test ≤ P95 as low risk; > P95 as high risk. Factors with *p* < 0.05 at univariate survival analysis were calculated in the decision tree model, which had an average accuracy value of 92.83%. The decision tree model was constructed with four layers reflecting the importance of each factor in predicting the risk of causing an indigenous outbreak. The first layer was divided by VOC period, indicating that VOC period had the greatest influence, while the second layer of influencing factors was the entry/exit risk occupation, the third layer was the region, and the fourth layer was the COVID-19 vaccination doses.

**Figure 1:**
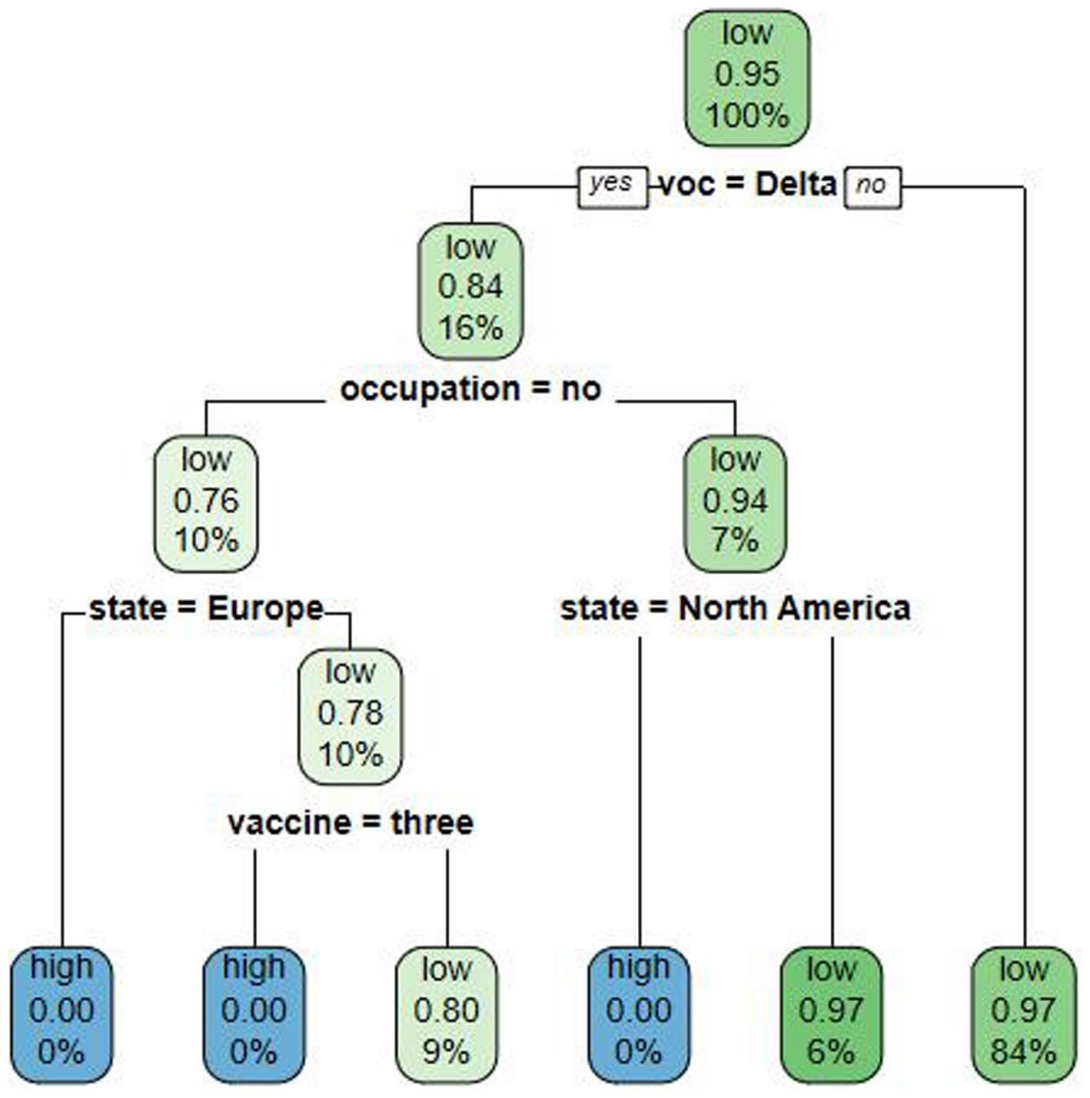
Multi-risk factor decision tree modeling of COVID-19 outbreak risk.

We found that the probability that an imported case during the Delta period causes an indigenous outbreak to be a low risk was 0.84, which gave a high-risk probability of 0.16, which was higher than in other periods such as the Omicron period. Among Delta-period inbound travelers, the probability of an entry/exit low-risk occupational case causing a high risk of an indigenous outbreak was 0.24, which was higher than that of an entry/exit high-risk occupational case (0.06). As Europe and North America were nonsignificant factors in the univariate analysis, the model did not predict risk for these cases. The orientation of the factors influencing the risk of an indigenous outbreak resulting from imported cases, as revealed by the decision tree analysis, is in line with the orientation of the factors influencing the duration of the time interval between entry and the first positive nucleic acid test, as determined by the multivariate analysis (Figure 1).

## Discussion

This paper provided a new estimation of the incubation period for COVID-19, focusing only on the period from entry to the time when the organism is infectious, expressed as the interval between the entry of an infected person and the time point when a positive test can be detected for the first time in a consecutive nucleic acid test. Moreover, the epidemiologic history of patients can be reproduced naturally based on interval-censored data (16).

The quarantine period of an infectious disease is generally the longest incubation period of the infectious disease (17). This paper estimated from a Log-normal distribution (18) that the approximate 95th percentile (94.51%) of the time interval between entry and the first positive nucleic acid test was 14 days, which is consistent with the conventional quarantine period of 14 days (19). Quarantine of 95% of the inbound travelers can effectively control the spread of the disease and prevent an epidemic on a large scale (20, 21). This study found that the time interval between entry and the first positive nucleic acid test for the total imported case was comparable to that of the Omicron period, so was the quarantine policy of the Omicron period utilized as a reference? Research had concluded that shortened quarantine period was attributed to the shorter average incubation period of the disease in the Omicron period (22, 23), milder clinical manifestations (24, 25), and better prognosis in most populations. As the epidemiological characteristics of the imported cases in this study varied, the quarantine policy during the Omicron period may not be suitable for the actual situation in this study. Therefore, selecting an appropriate quarantine period requires assessing the infectiousness and pathogenicity of the infectious disease at the time, as well as its societal risks.

Multivariate analysis found that the time interval between entry and the first positive nucleic acid test was shorter for people from Africa, entry/exit high-risk occupations, and people who were unvaccinated. The reason for this may be the low coverage of healthcare systems and low vaccination rates in Africa (26), consistent with the entry/exit high-risk occupations and those who have not been vaccinated with the COVID-19 vaccine, which have high viral loads in their bodies and are more likely to be detected during the quarantine period and immediately transferred for treatment, and therefore the risk of causing an indigenous outbreak is low. In contrast, those with longer positive detection intervals, such as those in entry/exit low-risk occupations, those who were fully vaccinated, and those who came from Asia, who appear to be “low-risk groups,” have a relatively lower rate of positive detections during the quarantine period, and may become a potential infection source after quarantine period, which may result in a higher risk of causing an indigenous outbreak (27). Overall, it was found that the “low-risk group” should be given more attention than the “high-risk group.” In addition, CART found that low-risk occupations only affected the imported cases of Delta strains, but had no significance on the imported cases of other strains. This suggested that there should be more detailed records for imported Delta cases. The risk of causing an indigenous outbreak significantly increased when Delta and low-risk occupations were present in imported cases. Therefore, it is recommended to strengthen the degree of tracking of these persons after the end of the quarantine period.

Facing the potential emergence of various infectious diseases, it is imperative to construct a precise, cost-effective epidemic prevention and control system that promotes comprehensive economic and social development at the lowest possible cost, provided that there is a high probability of successfully identifying potentially infected individuals. This study proposes the development of a risk-graded management model for inbound travelers, which can effectively track the follow-up situation of inbound risky travelers with longer positive test times through big data after the end of the quarantine period. By improving the accuracy and efficiency of case detection, this approach can help minimize the risk of indigenous epidemic transmission caused by imported cases.

Some limitations of this study should be noted. First, the data collected in this study were partially derived from interviews with imported infected persons, which is subject to potential recall bias. Second, this study did not include all imported COVID-19 cases and therefore cannot represent the epidemiologic characteristics of all imported cases in Guangzhou. Third, the time range of the imported cases included in this study was relatively wide. If there is a sufficient sample size of inbound cases, a study should have chosen data from a short period of time to speculate on the quarantine period, which will be more accurate.

## Conclusion

We found that those “low-risk groups” with low-risk occupations, fully vaccinated, and who came from Asia have a higher risk of developing an indigenous outbreak, and should be given more attention than the “high-risk groups.” It is recommended to keep tracking these “low-risk groups” after the end of the quarantine period, to achieve precise prevention and control. This underscores the need to identify the risk groups through influencing factors, which can inform emerging infectious diseases.

## Data availability statement

The datasets presented in this article are not readily available because due to contractual agreements with the data provider, the data used to produce this manuscript are not available for distribution. The analytic code is available upon request to the corresponding author. Requests to access the datasets should be directed to WZ, 1137164682@qq.com.

## Ethics statement

The studies involving humans were approved by Institutional Review Board of the Guangzhou Center for Disease Control and Prevention. The studies were conducted in accordance with the local legislation and institutional requirements. The ethics committee/institutional review board waived the requirement of written informed consent for participation from the participants or the participants' legal guardians/next of kin because this trial is a retrospective study, only collecting patients' clinical data, does not affect any medical rights of patients, and does not additionally increase the risk of patients. We only analyze the sample data and all are implemented through sample coding to protect the privacy of the subjects fully.

## Author contributions

WZ: Data curation, Writing – original draft. YT: Writing – original draft. ZZ: Writing – original draft. JC: Writing – original draft. XD: Writing – review & editing. XC: Writing – review & editing.
