# Supplementary material for: “Low-risk groups” deserve more attention than “high-risk groups” in imported COVID-19 cases
Source: Front Med (Lausanne). 2023 Nov 30;10:1293747. doi: 10.3389/fmed.2023.1293747 (PMC10720434; doi:10.3389/fmed.2023.1293747)
Supplement: Supplementary file 1 [file Table_1.docx]

Table S1. Univariate survival analysis of factors influencing the time interval between entry and the first positive nucleic acid test of COVID-19

| Variable category |  | References | B | SE | **2 | p values | OR |
| --- | --- | --- | --- | --- | --- | --- | --- |
| Sex | men | women | 0.05 | 0.21 | 0.05 | 0.83 | 1.05 |
| Age | 18~ | <18 | -0.20 | 0.41 | 0.24 | 0.63 | 0.82 |
|  | 40~ |  | -0.23 | 0.41 | 0.31 | 0.58 | 0.79 |
|  | ≥65 |  | 0.02 | 0.54 | 0.00 | 0.97 | 1.02 |
| Entry/exit risk occupations | high | low | -1.22 | 0.12 | 27.11 | <0.0001 | 0.30 |
| Region | North America | Asia | 0.25 | 0.45 | 0.30 | 0.59 | 1.28 |
|  | Oceania |  | 0.82 | 0.34 | 5.68 | 0.02 | 2.27 |
|  | Africa |  | -1.79 | 0.40 | 19.75 | <0.0001 | 0.17 |
|  | South America |  | -0.58 | 1.53 | 0.14 | 0.71 | 0.56 |
|  | Europe |  | 0.11 | 0.51 | 0.04 | 0.84 | 1.12 |
| Nationality | Hong Kong, Macau, and Taiwan | Chinese | -0.91 | 1.41 | 0.42 | 0.52 | 0.40 |
|  | Foreign |  | -0.16 | 0.22 | 0.55 | 0.46 | 0.85 |
| International student | yes | no | 0.43 | 0.30 | 2.02 | 0.15 | 1.54 |
| background diseases | unknown | no | 0.39 | 0.26 | 2.23 | 0.14 | 1.48 |
|  | yes |  | 0.14 | 0.43 | 0.11 | 0.74 | 1.15 |
| Vaccination doses | 1 dose | unvaccinated | 0.70 | 0.39 | 3.17 | 0.07 | 2.01 |
|  | 2 doses |  | 1.24 | 0.24 | 25.67 | <0.0001 | 3.46 |
|  | 3 or more doses |  | 1.31 | 0.25 | 27.91 | <0.0001 | 3.71 |
| VOC periods | Alpha | Delta | -3.15 | 0.34 | 84.24 | <0.0001 | 0.04 |
|  | Beta |  | -5.14 | 1.08 | 22.58 | <0.0001 | 0.01 |
|  | Gamma |  | -1.68 | 1.07 | 2.46 | 0.12 | 0.19 |
|  | Omicron |  | -0.89 | 0.23 | 14.96 | 0.0001 | 0.41 |
